# Threatened species drive the strength of the carbonate pump in the northern Scotia Sea

**DOI:** 10.1038/s41467-018-07088-y

**Published:** 2018-11-02

**Authors:** C. Manno, F. Giglio, G. Stowasser, S. Fielding, P. Enderlein, G. A. Tarling

**Affiliations:** 1 British Antarctic Survey, Natural Environment Research Council, Cambridge, CB3 0ET UK; 2 CNR-Ismar (Institute of Marine Science), Via P. Gobetti 101, 40129, Bologna, Italy

## Abstract

The efficiency of deep-ocean CO_2_ sequestration is regulated by the relative balance between inorganic and organic carbon export respectively acting through the biological carbon pump (BCP) and the carbonate counter pump (CCP). The composition and abundance of calcifying species in the prevailing oceanic plankton community plays a major role in driving the CCP. Here we assess the role of these calcifying organisms in regulating the strength of the CCP in a Southern Ocean region (northern Scotia Sea) known to be a major hotspot for the drawdown of atmospheric CO_2_. We show that, when shelled pteropods dominate the calcifying community, the total annual reduction of CO_2_ transferred to the deep ocean doubles (17%) compared to when other plankton calcifiers dominate (3–9%). Furthermore, predation enhances their contribution through the removal of organic soft tissue. Pteropods are threatened in polar regions by ocean warming and acidification. We determine that their potential decline would have major implications to the comparative strengths of the BCP and CCP.

## Abstract (web summary)

The Scotia Sea, located in the Southern Ocean, is a major hotspot for the drawdown of atmospheric CO_2_. Here, the authors show that the strength of the carbonate counter pump doubles when shelled pteropods dominate the plankton calcifier community, counteracting the amount of CO_2_ transferred to the deep ocean.

## Introduction

The biological carbon pump (BCP) is the process that draws down atmospheric carbon dioxide (CO_2_) through the fixation of inorganic carbon by photosynthesis and the consequent export and sequestration of particulate organic carbon (POC) to the deep ocean^1^. The BCP is counteracted by the carbonate counter pump (CCP), which causes an increase in surface ocean CO_2_ through the calcification and precipitation of carbonate and the resulting export of particulate inorganic carbon (PIC). In order to determine the efficiency of deep-ocean CO_2_ sequestration and, in turn, how this may affect the concentrations of CO_2_ in the surface ocean, it is crucial to understand the dynamics of the export of both PIC and POC, particularly their relative balance, termed the PIC:POC or rain ratio^2^.

A number of biological processes affect these export dynamics, with zooplankton playing an important role. For instance, the fate of exported POC can be influenced through zooplankton grazing, repackaging of organic matter into faecal pellets (FP), and the vertical migration and transport of carbon and nutrients into the mesopelagic zone (e.g., ^3,4^).

Calcifying zooplankton such as pteropods, ostracods and foraminifera can influence the PIC flux through removing calcium (Ca^2+^) from the pelagic surface ocean waters to form shells and structures of calcium carbonate (CaCO_3_). These calcifiers play an important role in PIC sequestration to the deep ocean because their relatively large size and high density makes them sink rapidly^5,6^. By comparison, calcifying phytoplankton such as coccolithophores have slower sinking rates but their sedimentation flux can be accelerated through assimilation into larger biological aggregates such as zooplankton FP^7^. Thus, the level of carbonate precipitation, as well as the ability to act as ballast, depend on the composition of calcifying species within the plankton community^8,9^.

Despite understanding variability in the relative abundance and composition of calcifying species is a crucial part of accounting for the fate of CO_2_ uptake and sequestration, little work has been done specifically examining seasonal dynamics of calcifying communities.

In the Southern Ocean (SO), calcifying organisms are often very abundant and can dominate carbonate fluxes^10–12^ as well as the biogeochemical stoichiometry of the particle flux component^13,14^.

Here, we describe a 2-year time series of particle flux, as measured by two deep moored sediment traps (P2 and P3) located in the SO (northern Scotia Sea sector), a globally important region of atmospheric CO_2_ drawdown^15^ containing both naturally iron-fertilised (P3) and iron-limited (P2) regimes. Previously, we have shown how the POC flux in this region is mainly driven by zooplankton FC^16,17^ as well as diatoms^12^. However, the relative contribution of calcifying organisms to deep carbonate export and their regulation of the PIC:POC ratio has yet to be determined in this region. The aim of this study was to assess the seasonal and regional change in the deep carbonate flux. The specific contribution of each part of the plankton calcifying community (pteropods, foraminifera, coccolithphores and ostracods) to the strength of the carbonate pump was also investigated. We discriminated between the carbonate contributions of predated (empty shells) and non-predated (shell with the soft body) pteropods and between individual and aggregate (included in FP) coccoliths so as to quantify the intensity of the different carbonate pump routes mediated by different food-web process. This work allows us to define the importance of the calcifying plankton community to determining the magnitude of the CCP. The calcifying community in the SO is nevertheless particularly under stress from the intensification of ocean acidification (OA) because of the higher solubility of CO_2_ in cold water and the naturally low levels of carbonate ion concentration in this region^18^. The sensitivity of the CCP to forecast OA levels is therefore also a matter of concern that we give consideration to in the Discussion.

**Table 1:** Seasonal and annual counter carbonate pump effect (CCP) at sediment traps P2 and P3.

	%CCP				
	Total CaCO_3_	Ptero	Cocco	Fora	Ostrac
**P2**					
Autumn	3.62	1.20	0.52	1.82	0.07
Winter	1.35	0.16	0.06	1.11	0.16
Spring	2.35	0.36	1.40	0.55	0.05
Summer	4.16	1.31	1.10	1.71	0.04
Autumn	21.80	14.72	3.64	3.31	0.24
Winter	14.91	3.85	3.28	3.28	4.65
Spring	14.94	4.68	5.99	3.22	0.10
Summer	19.40	11.62	5.41	2.54	1.37
2009	3.36	0.96	0.94	1.42	0.06
2010	17.57	9.20	4.55	3.12	0.90
**P3**					
Autumn	6.97	1.07	1.67	4.02	0.21
Winter	11.08	1.15	1.36	8.35	0.22
Spring	16.43	5.02	9.09	2.01	0.33
Summer	4.48	1.14	1.58	1.72	0.04
Autumn	21.91	12.12	2.72	7.06	0.00
Winter	14.42	0.48	0.72	3.8	8.42
Spring	18.05	6.2	10.27	1.22	0.36
Summer	12.70	6.14	5.25	1.31	0.00
2009	9.49	2.64	4.50	2.19	0.17
2010	16.67	8.01	4.04	4.12	0.49

This represents the reduction (%) of CO_2_ transferred to the deep ocean due to the production of total carbonate. The contribution of each calcifying organism (pteropods, coccolithophores, foraminifera and ostracods) to the CO_2_ reduction is also calculated

## Results

### Calcifier community contribution to the carbonate flux

**Fig. 1:**
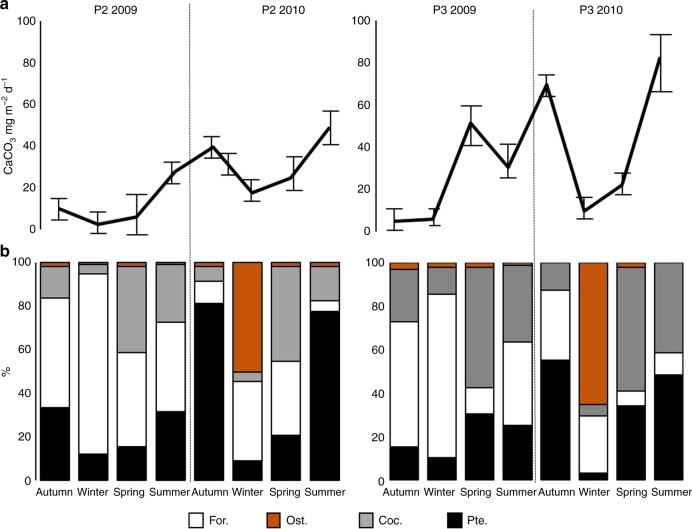
Seasonal carbonate flux. **a** Seasonal carbonate flux (CaCO_3_) expressed as mg m^−^^2^ d^−1^; **b** contribution of planktonic calcifiers (%) to the total CaCO_3_ flux, (pteropods, foraminifera, coccolithophores and ostracods) at the two deep moored sediment traps P2 and P3 for each year (2009 and 2010). Error bars indicate 1 standard deviation (replicates *n* = 8–12)

Total carbonate (CaCO_3_) flux showed a strong temporal variability, with values ranging from 2.15 to 48.49 mg m^−2^ d^−1^ and from 4.78 to 81.89 mg m^−^^2^ d^−1^ at P2 and P3, respectively. Peak carbonate flux was reached at both sites in 2010 with maximum values in autumn and the following summer (Fig. 1a). In general, carbonate flux was higher in 2010 compared to 2009, but significantly higher only at P2 (*p* ≤ 0.05, *Z* = −3.0594, *p* = 0.00222). The relative contribution of the calcifying plankton community to total carbonate flux significantly changed between seasons and years (Fig.1b) (*p* ≤ 0.05, *Z* = −3.0596, *p* = 0.00223). However, the maximum carbonate flux in 2010 corresponded with periods when pteropod shells significantly dominated the calcifying plankton community (with respect to CaCO_3_) compared to 2009 (up to 83% at P2 and up to 55% at P3 in autumn and then summer 2010). Conversely, foraminifera significantly dominated the contribution to the autumn and summer carbonate fluxes (up to 57%) in 2009 compared to 2010, although carbonate flux was comparatively lower. Coccolithophores dominated the spring carbonate flux in both years and at both sites (up to 56%). Ostracods generally made a low contribution to the carbonate flux with the exception of winter 2010, when they were the dominant contributors (65%).

**Fig. 2:**
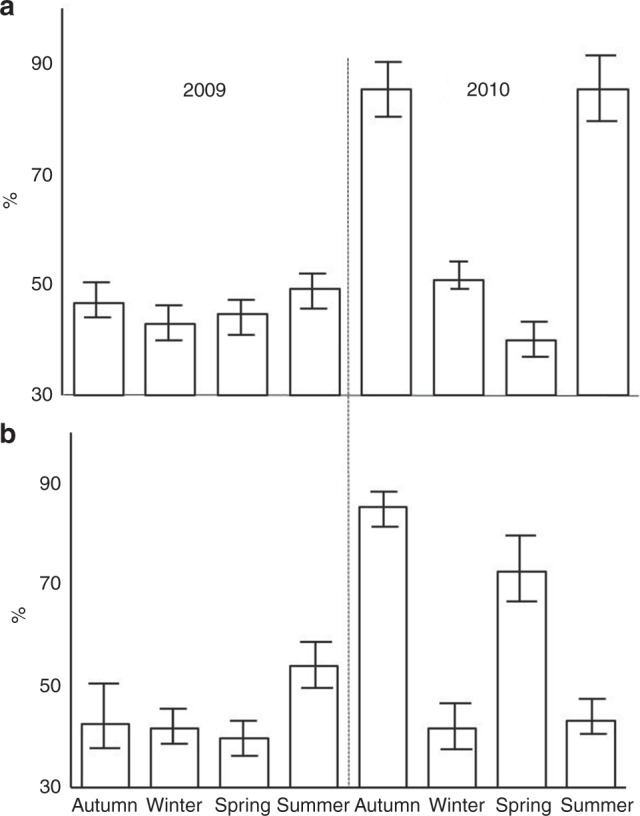
Empty pteropod shells. Empty pteropod shells expressed as seasonal % of the total pteropods collected for each year (2009–2010) at P2 (**a**) and P3 (**b**). Error bars indicate 1 standard deviation (replicates *n* = 4–6)

**Fig. 3:**
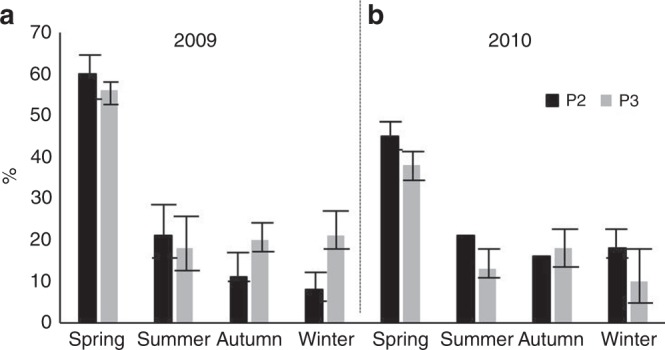
Coccolithophore included within faecal pellets. Coccolithophore aggregates included within faecal pellets, expressed as seasonal contribution (%) of the total coccolithophore assemblage for each year **a** 2009 and **b** 2010 at P2 and P3. Error bars indicate 1 standard deviation (replicates *n* = 4–6)

The presence of pteropods with empty shells significantly changed between years (*Z* = −2.0896, *p* = 0.03662). In 2009, the majority of pteropod shells at both sites contained soft body tissue (an average of >50% across all seasons, Fig. 2). Conversely, in the autumn and summer of 2010, up to 85% of pteropod shells were empty. Overall, coccolithophores were mainly found as single cells however, in spring, the fraction aggregated into FP at both sites were significantly higher (*Z* = −2.1245, *p* = 0.03236), particularly at P3 in 2009, when up to 60% of coccolithophores were found within FP material (Fig. 3).

Carbonate flux and calcifying plankton community composition data are provided in Supplementary Data 1.

### BSi:PIC:POC ratio and carbonate counter pump effect

**Fig. 4:**
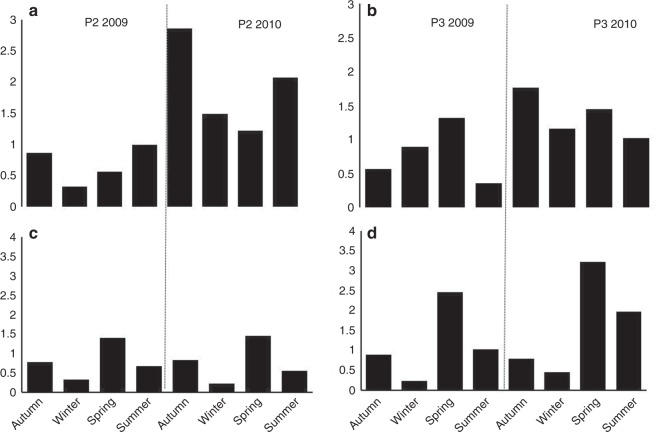
Variability of the stoichiometric ratio. Seasonal variability of the stoichiometric ratio of PIC: POC at P2 (**a**) and P3 (**b**) and BSi:PIC at P2 (**c**) and P3 (**d**) for each year (2009 and 2010)

A PIC:POC ratio greater than 1 is indicative of export dominated by calcifiers. PIC:POC ratio was significantly (*p* ≤ 0.05) different between 2009 and 2010 at both sites (P2, *Z* = −3.0594; *p* = 0.00222; P3, *Z* = −2.2228, *p* = 0.02642). In 2009, the PIC:POC ratio was <1 over most of the seasonal cycle at both sites except in spring at P3 (1.32, Fig. 4). In 2010, PIC:POC was always >1, with maximum values in autumn (2.07) and summer (2.85) at P2. A BSi:PIC ratio greater than 1 is indicative of diatom-dominated export. The BSi:PIC ratio was significantly (*p* ≤ 0.05) different between 2009 and 2010 at P3 (*Z* = −2.0396, *p* = 0.04136). The BSi:PIC was <1 at both stations in both years during autumn and winter (ranging from 0.23 in winter to 0.83 in autumn). During the spring, BSi:PIC was always >1 with maximum values found at P3 in 2010 (3.22). Refer to Supplementary Data 1 for BSi, PIC and POC flux data.

The total annual reduction of CO_2_ transferred to the deep ocean due to the production of carbonate (%CCP, Table 1) was significantly different between sites and years (*Z* = −2.8031, *p* = 0.00512). In particular it was stronger during 2010 (up to 17%) than 2009 (up to 9%). Pteropod carbonate was the main contributor to the high %CCP values found in 2010 while summed foramanifera and coccolithiphore carbonate dominated the lower %CCP of 2009. Refer to Supplementary Data 2 for full calculation of CCP.

## Discussion

We found that the magnitude of the CCP was influenced by both seasonal and interannual variability in the calcifying plankton community. Pteropods were the main producer of CaCO_3_ in this region and were the major driver of variations in the carbonate flux. In particular, when pteropods dominated the calcifying community in 2010, the total annual reduction of CO_2_ transferred to the deep ocean was an order of magnitude higher in both study sites compared to the previous year when foraminifera and coccolithiphores dominated. The contribution of ostracods to carbonate flux was relatively minor through most of the year, but became important during the winter, underlining the importance of including these organisms in carbonate flux calculations.

Our finding of the major role of pteropods in driving a powerful CCP is significant because, in the SO, this organism can comprise a large component of the zooplankton community^19,20^. The Northern Scotia Sea, Weddell Sea and the Ross Sea are hotspots in terms of both pteropod densities and their proportional contribution to the total zooplankton^20,21^. In the SO, pteropod population densities can experience high interannual variability mainly as a result of variation in primary production^22^ and complex population dynamics involving overlapping generations^23^.

Over an annual cycle in the Crozet region of the SO it was estimated that the production of foraminifera dominated the flux of carbonate in naturally iron-fertilised waters and reduced the overall amount of CO_2_ transferred to the deep ocean by up 8 times compared to a neighbouring non-fertilised site^24^. In the present study, we show that the level of interannual variability in the calcifying plankton community (i.e., foraminifera dominated in 2009 vs. pteropod dominated in 2010) can have a larger impact on the CCP than regional variability between iron fertilised and non-fertilised sites. For instance, even though the CCP was three times higher in the iron fertilised P3 site (9%) compared to the non-fertilised P2 site (3%), in agreement with the Crozet region study of Salter et al.^24^, this was not the case in 2010 when, as a result of the dominance of pteropods, CCP in P2 was similarly high in P3 (17%). This illustrates that that the CCP itself is strongly influenced by changes in community structure between years.

We identified geochemical signatures associated with the different calcifiers that drive the CCP. Specifically, signatures where BSi:PIC > 1 and PIC:POC > 1 were commonly associated with the coccolithophorid contribution to the CCP (although not the case in spring 2009 at P2), while signatures where BSi:PIC < 1 and PIC:POC > 1 indicated a major contribution from pteropods (with the exception of autumn 2010 at P3). Salter et al.^24^ suggested that BSi:PIC > 1 and PIC:POC > 1 were unique characteristics of the iron-fertilised region of the polar frontal zone. We show, otherwise, that the combined geochemical signature within the iron-fertilised region can differ between years as a result of interannual variability in calcifying assemblages. The similar bloom conditions in both years (Supplementary Fig. 1) support the view that calcification was primarily responsible for the observed variation in the BSi:PIC:POC geochemical signature

**Fig. 5:**
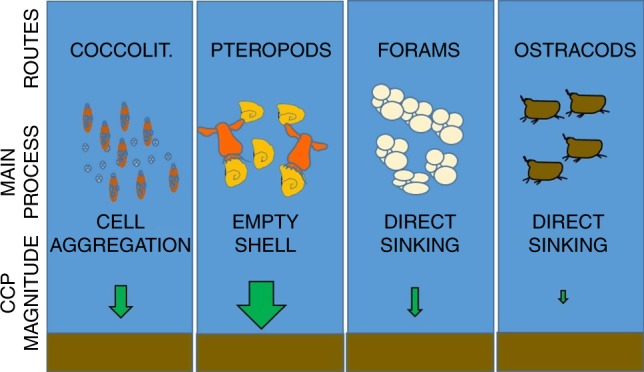
Dominant process driving the carbonate counter pump. Schematic diagram highlighting the dominant process driving the carbonate counter pump during the different seasons in the Scotia Sea

A further major influence on the magnitude of the CCP are food-web processes within the calcifying plankton community, which can affect the CCP through a number of different routes (Fig. 5). In particular, a combination of predation and zooplankton grazing can have a large influence on the efficiency of the CCP. For instance, coccolithophores are mostly dominant during the spring but they can strongly contribute to the CCP (up to 10%) when mainly aggregated in FP, as seen at P3 when up to 60% of coccolithophores in deep sediment traps were in this form. By contrast, where coccolithophores in the deep traps were mainly found as single cells, as in P2, their contribution to the CCP was much lower (2%). The important role of FP as drivers of POC export in the Scotia Sea was highlighted by Jones et al. and Manno et al.^15,16^. Here, we demonstrate that FP are also important drivers of PIC export. Zooplankton attain high levels of biomass at station P3, and their generation of large levels of FP provides a major conduit for coccolithophore export during the late spring–early summer. Ballast provided by coccolithophore mediated carbonate significantly increase the sinking speed of FP produced by key zooplankton organisms such copepods which feed in part on coccolithophorid diets^25^.

In 2010, pteropods dominated the calcifying plankton community in summer and autumn at both sites. However, their overall contribution to the CCP appeared to depend on whether shells were empty or included soft tissue. Where empty shells predominate, such as in 2010 (at both stations during autumn and summer) when they contributed up to 85% of the pteropod community in the sediment traps, their contribution to the CCP was as high as 15% compared to when shells with soft tissue predominated and their CCP contribution was just 7%. Empty shells are generated through predation activity by gymnosomatous (non-shelled) pteropods such as *Clione* spp. These feed almost exclusively on shelled pteropods, using tentacles to grab the shell and extract the soft body tissue. Without any soft body tissue, these shells are almost completely devoid of POC and therefore mainly contribute PIC to the downward flux on sinking. Conversely, when soft body tissue is present, the sinking pteropods contribute both POC and PIC to the downward flux. Assuming a shell and soft body contribution to the total dry biomass of 70% and 30%, respectively^26^ we can estimate an increase in contributions to the CCP of up to 26% (full vs. empty shells). During the late summer–early autumn, a combination of environmental and behavioural factors act to promote a high pteropod sinking flux^10,11,26^ which, when combined with the predatory activity by *Clione* spp., can represent a substantial input of PIC to the deep ocean.

We show that the role of calcifying zooplankton in regulating the strength of the CCP is a function of the variability in the calcifying plankton community and associated food-web processes. Rising ocean temperatures, decreasing oxygen, OA, eutrophication, overfishing, and species introductions are changing plankton communities, and synergistic effects of these factors is expected to alter carbon cycling by zooplankton and to have direct impacts on key species^27^. Furthermore, carbonate undersaturation events have been predicted to spread rapidly^28^, affecting 70% of the SO by 2100 with the duration of these events increasing abruptly to up to 6 months per year in less than 20 years. SO zooplankton, such as pteropods, have comparatively long life-cycles and limited numbers of generation cycles in which to adapt to such rapid changes^29^. For instance, in foraminifera, it has been shown that shell weight decreases when carbonate ion levels are lowered^30,31^, while coccolithophore aggregates are less likely to form in simulated OA conditions^32^, reducing their potential to sink. However, amongst all planktonic calcifiers, it is the pteropods that are likely to be most sensitive to the change in seawater chemistry^33–36^ because of their aragonite shell structure, the relatively more soluble form of CaCO_3_^37^. Thus, within SO regions where the CCP is dominated by pteropods, such as the Northern Scotia Sea, there are likely to be dramatic changes in levels of direct PIC sinking and the ballasting of all particulate matter as a result of OA. We demonstrate the fundamental role that pteropods play in the CCP within a major area of carbon drawdown in the SO^15^. This emphasises the even greater urgency of parameterising the consequences of the potential declines in these organisms to the wider Earth system.

## Methods

### Sample collection

Bottom-tethered moorings were repeatedly deployed at two locations (SCOOBIES sites P2 and P3) for periods of approximately 24 months between April 2009 and March 2010 (labelled 2009) and April 2010–February 2011 (labelled 2010). P2 was located at a site that was oceanographically upstream of South Georgia (55° 11.99′S, 40° 07.42′W), while P3 was downstream (52° 43.40′S, 40° 08.83′W). Refer to Supplementary Fig. 2 for mooring position. Each sediment trap (McLane Parflux sediment traps, 0.5 m^2^ surface collecting area; McLane Labs, Falmouth, MA, USA) carried 21 receiving cups and was fitted with a plastic baffle mounted in the opening, to prevent the entrance of large organisms. Prior to deployment, the receiving cups were filled with NaCl buffered HgCl_2_ seawater solution to arrest biological degradation during sample collection and to avoid carbonate dissolution. Traps were deployed at a depth of 1500 m (P2, water depth 3200 m) and 2000 m (P3, water depth 3800 m), and the sample carousel was programmed to rotate at intervals of 15 days in austral summer and 30 days in austral winter. Note that physical data acquired during the sediment trap deployment suggest the record was not subjected to major hydrodynamic biases since mean current velocities near to the sediment traps were <10 cm s^−1^ and we assumed no significant lateral advection occurred.

### Trap sample processing

Once in the laboratory, the supernatant of each cup was removed by pipette and its pH was measured in order to check for possible carbonate dissolution. Prior to splitting, swimmers, i.e., zooplanktonic organisms that can enter the receiving cups while alive, were carefully removed: samples were first wet-sieved through a 1 mm nylon mesh and the remaining swimmers were hand-picked under a dissecting microscope. Large aggregates, fragments of moults and empty tests retained by the mesh were returned to the sample. Each sample was then divided into a series of replicate fractions for subsequent analysis using a McLane rotary sample splitter (McLane Labs, Falmouth, MA, USA).

### Biogeochemistry analysis

Replicate fractions were vacuum filtered through pre-weighing and pre-combustion (550 °C for 5 h) on Whatman GF/F filters. Filters were then desalted through briefly washing with distilled water and dried at 60 °C. POC was measured by combustion in an elemental analyser (CHN); for POC determination, filters were pre-treated with 2 N H_3_PO_4_ and 1 N HCl. A detailed description of POC sample processing, analyses and results are provided in Manno et al. (2015). To estimate carbonate (CaCO_3_) particle fluxes, PIC was obtained by determining the difference between total particulate carbon and POC and multiplied by a factor of 8.33, assuming that all inorganic carbon was in the form of calcium carbonate. CaCO_3_ and PIC flux was expressed in mg m^−^^2^ d^−^^1^, estimated by dividing the total mass per sample by the time interval and the trap collection area. Biogenic silica (BSi) was determined following a progressive dissolution method^38^, followed by colorimetric analysis. NaOH 0.5 M was used as extractant, in view of the large concentrations of biogenic silica in the samples. An aliquot of 0.2 ml was taken for silicic acid analysis every hour and the BSi content was estimated from the intercept of the extraction timeseries^38^.

### Calcifying plankton component

Pteropods, ostracods and foraminifera were counted and picked by using a light microscope (Olympus SZX16) from sub-sample aliquots. In the case of pteropods, we discriminated between pteropods containing soft body and empty shells. We assumed that empty shells were the product of successful attacks by their main predator *Clione limacina*. Ostracods were all recovered with soft body inside. Ostracods and pteropods with pristine, transparent shells were collected but not included in the carbonate calculation because they were considered as swimmers^16^. CaCO_3_ was measured for each taxonomic group. Pteropods, foraminifera and ostracods picked from the samples were rinsed with distilled water and air dried for 24 h. For each sample, pteropods, foraminifera and ostracods were separately combusted in a muffle furnace at 550 °C for 5 h to remove any organic carbon. After combustion, the ash was air dried and weighed on a microbalance to estimate the total amount of carbonate in the sample for each taxonomic group. The coccolithophore carbonate amount was indirectly estimated by analysing the amount of CaCO_3_ left in the sample after the removal of pteropods, ostracod and foraminifera.

### Carbonate content of zooplankton FPs

Hundred FPs were picked from each sub-sample for carbonate analysis by using a light microscope. FP carbonate content was estimated through combustion in a muffle furnace as above to remove any organic material. The residual C (representing only the PIC fraction) was measured by CHN and then converted to CaCO_3_ as above. We assumed that coccolithophores were responsible for all measured carbonate. We verified this through inverted microscope analysis of FP selected randomly from across the full sample set. Foraminifera abundance within FP was always <4% of the total foraminifera abundance, while ostracods and pteropod shells were absent.

### Carbonate pump calculation

The strength of the carbonate counter pump, expressed as the reduction of the CO_2_ drawdown by the biological pump due to CO_2_ production during the calcification process in the mixed layer^39,40^ was calculated as follows:

1 $$({\mathrm{CC}}_{{\mathrm{pump}}},\,\% ) = \left( {{\mathrm{PIC}}_{{\mathrm{flux}}} \times {\mathit{\Psi }}} \right)/\left( {{\mathrm{POC}}_{{\mathrm{WLMflux}}} \times 100} \right){,}$$

where CC_pump_ is the % of carbonate counter pump effect; *Ψ* is the mole of CO_2_ emitted by a mole of CO_3_^2+^ precipitated during the calcification process and ranges from 0.7 to 0.8 (we used 0.75 as average value) for seawater between 2 and 5 °C and a pCO_2_ of 300–400 atm;^39^ PIC is the CaCO_3_ flux measured at the sediment trap (taken as a minimum estimate of PIC_flux_ at the base of the winter mixed layer); POC_WLMflux_ is the POC flux measured at the sediment trap where the deployment depth were normalised to the base of the winter mixed layer (200 m) using the expression: *F*_WML_ = *F*_d_ (WML/*d*)^*b*^; *F*_d_ is the flux at the sediment trap deployment depth, *d* is the sediment trap deployment depth, WML is 200 m and the exponent *b* characterises the attenuation of flux with depth. A *b* regional value of 1.43 and 0.78^41^ was used at P2 and P3, respectively.

### Statistical analysis

Wilcoxon signed-rank tests were used to determine whether there were any significant differences between sites and between seasons with regard to the pteropods, foraminifera, coccolithophores, ostracod, CaCO_3_ flux and the PIC:POC, BSi:PIC. Differences were considered significant where *α* < 0.05.

## Electronic supplementary material

Supplementary Information

Description of Additional Supplementary Files

Supplementary Data 1

Supplementary Data 2

## Data Availability

All published data set is available at NERC—Polar Data Centre, Cambridge, UK. DOI link: 10.5285/749ffa17-ee4f-46a4-8827-5f062af19a6c; Short DOI link: https://doi.org/cvfr; Short DOI: 10/cvfr
