# Supplementary material for: Threatened species drive the strength of the carbonate pump in the northern Scotia Sea
Source: Nat Commun. 2018 Nov 2;9:4592. doi: 10.1038/s41467-018-07088-y (PMC6214935; doi:10.1038/s41467-018-07088-y)
Supplement: Supplementary file 1 — Supplementary Information [file 41467_2018_7088_MOESM1_ESM.pdf]

**Supplementary Information**

**Threatened species drive the strength of the carbonate pump in the Scotia Sea (Southern Ocean)**

Manno et al.

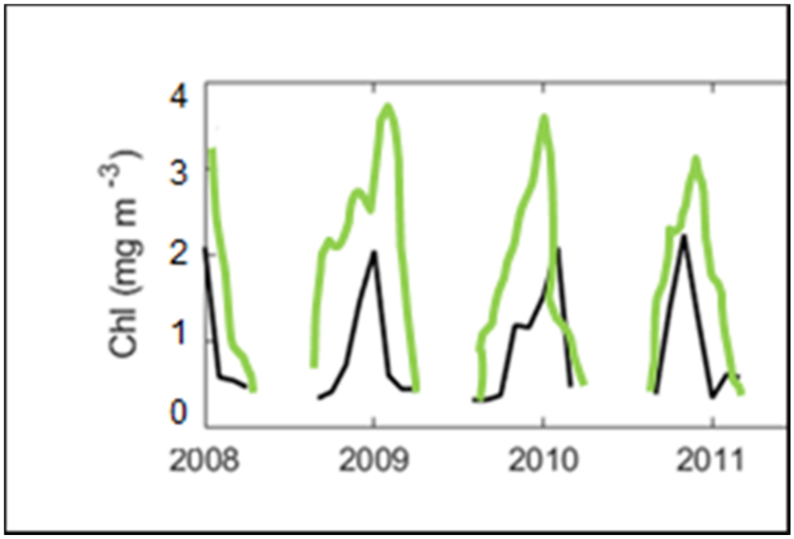


**Supplementary Fig. 1:** Monthly mean satellite chlorophyll a (Chl) concentration (mg m-3) at the P2 (Black line) and P3 (green lines) site for the period 2008-2011. Data have been averaged over a 55 x 55 km box centred on the P2 and P3 site.

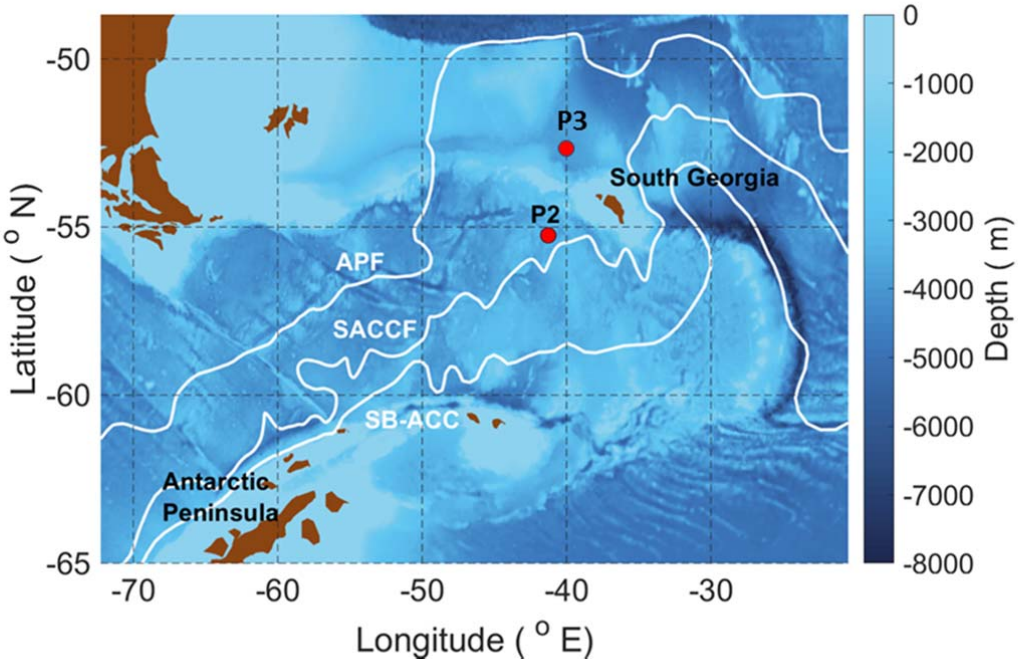


**Supplementary Fig. 2:** Location of sediment trap mooring (P2 and P3) in the Scotia Sea, Southern Ocean. White lines display mean frontal positions of the Antarctic Polar Front (APF), Southern Antarctic Circumpolar Current Front (SACCF) and the Southern Boundary-Antarctic Circumpolar Current (SB-ACC) Bathymetry data from ETOPO1 Global relief model. The figure is provided by Anna Belcher (British Antarctic Survey)
