# Supplementary material for: Threatened species drive the strength of the carbonate pump in the northern Scotia Sea
Source: Nat Commun. 2018 Nov 2;9:4592. doi: 10.1038/s41467-018-07088-y (PMC6214935; doi:10.1038/s41467-018-07088-y)
Supplement: Supplementary file 2 — Description of Additional Supplementary Files [file 41467_2018_7088_MOESM2_ESM.pdf]

Description of Additional Supplementary Files

File Name: Supplementary Data 1
Description: Biogeochemical particle flux monthly average (Bsi, Biosilica; POC, Particle Organic Carbon; PIC, Particle Inorganic Carbon) and zooplankton calcifies flux (PT, Pteropods; FOR, Foraminifera; COCC, Coccolithophores; OST, Ostracodes) expressed as mg*m-2*d-1, at P2 and P3 during the austral summer 2009/2010 and 2010/2011.

File Name: Supplementary Data 2
Description: Supplementary Data 2 Calculation of reduction of the CO2 drawdown by the biological pump due to CO2 production during the calcification process expressed as (CCpump,%)=(PICflux*Ψ)/POCWLMflux*100 , where : Ψ , mole of CO2 emitted by a mole of CO32+ precipitated during the calcification process; PIC is the CaCO3 flux measured at the sediment trap (taken as a minimum estimate of PIC flux at the base of the winter mixed layer); POCWLM flux is the POC flux measured at the sediment trap where the deployment depth were normalized to the base of the winter mixed layer (200 m) using the expression: FWML=Fd (WML/d)b; Fd is the flux at the sediment trap deployment depth, d is the sediment trap deployment depth, WML is 200m and the exponent b characterizes the attenuation of flux with depth in P2 and P3.
