# Supplementary material for: Threatened species drive the strength of the carbonate pump in the northern Scotia Sea
Source: Nat Commun. 2018 Nov 2;9:4592. doi: 10.1038/s41467-018-07088-y (PMC6214935; doi:10.1038/s41467-018-07088-y)
Supplement: Supplementary file 3 — Supplementary Data 1 [file 41467_2018_7088_MOESM3_ESM.docx]

**Supplementary Data 1**

**Threatened species drive the strength of the carbonate pump in the Scotia Sea (Southern Ocean)**

Manno et al.

|  |  | mg/m2/d |  |  |  |  |  |  |
| --- | --- | --- | --- | --- | --- | --- | --- | --- |
| P2 |  | Bsi | POC | PIC | PT | FOR | COCC | OST |
| 2009/2010 | APR | 0.52 | 1.34 | 0.90 | 2.27 | 3.92 | 0.93 | 0.15 |
|  | MAY | 1.05 | 1.36 | 1.33 | 4.14 | 5.39 | 1.71 | 0.47 |
|  | JUNE | 0.05 | 1.09 | 0.41 | 0.50 | 2.97 | 0.14 | 0.41 |
|  | JULY | 0.07 | 0.68 | 0.15 | 0.10 | 0.98 | 0.07 | 0.20 |
|  | AUG | 0.14 | 0.66 | 0.43 | 0.55 | 2.75 | 0.11 | 0.37 |
|  | SEP | 0.75 | 1.13 | 0.61 | 0.88 | 1.19 | 3.29 | 0.18 |
|  | OCT | 0.85 | 1.94 | 0.46 | 0.61 | 1.03 | 2.11 | 0.09 |
|  | NOV | 0.16 | 0.59 | 0.35 | 0.40 | 6.49 | 1.73 | 0.03 |
|  | DEC | 5.17 | 3.93 | 3.86 | 12.03 | 14.67 | 9.71 | 0.96 |
|  | JAN | 1.42 | 2.56 | 3.15 | 8.19 | 10.84 | 7.02 | 0.15 |
|  | FEB | 0.54 | 3.34 | 2.24 | 4.72 | 7.23 | 4.39 | 0.25 |
|  | MAR | 2.06 | 1.50 | 3.68 | 23.78 | 3.13 | 2.48 | 0.82 |
| 2010./2011 | APR | 6.88 | 1.40 | 5.14 | 37.86 | 5.33 | 2.67 | 0.48 |
|  | MAY | 3.41 | 1.80 | 4.01 | 26.19 | 2.97 | 1.86 | 0.71 |
|  | JUNE | 0.25 | 1.45 | 2.10 | 1.34 | 6.93 | 0.78 | 8.68 |
|  | JULY | 1.07 | 1.77 | 3.13 | 2.96 | 8.44 | 0.87 | 15.05 |
|  | AUG | 0.28 | 0.98 | 1.21 | 0.90 | 3.66 | 0.55 | 4.44 |
|  | SEP | 3.14 | 1.53 | 2.34 | 4.02 | 3.94 | 11.00 | 0.50 |
|  | OCT | 1.90 | 2.45 | 2.13 | 3.26 | 5.03 | 9.02 | 0.22 |
|  | NOV | 9.34 | 3.25 | 4.02 | 8.20 | 8.54 | 17.92 | 0.66 |
|  | DEC | 2.91 | 3.45 | 8.07 | 53.11 | 4.26 | 12.26 | 2.32 |
|  | JAN | 2.92 | 2.22 | 2.97 | 17.92 | 0.85 | 3.61 | 0.36 |
|  | FEB | 0.81 | 2.77 | 6.78 | 42.67 | 2.58 | 8.10 | 1.32 |
| P3 |  |  |  |  |  |  |  |  |
| 2009/2010 | APR | 1.62 | 1.65 | 1.29 | 1.86 | 6.47 | 2.17 | 0.41 |
|  | MAY | 0.12 | 0.63 | 0.21 | 0.22 | 0.97 | 0.48 | 0.04 |
|  | JUNE | 0.06 | 0.79 | 0.63 | 0.54 | 4.01 | 0.65 | 0.08 |
|  | JULY | 0.15 | 0.74 | 0.58 | 0.60 | 3.32 | 0.74 | 0.11 |
|  | AUG | 0.30 | 0.86 | 0.89 | 0.64 | 5.90 | 0.76 | 0.16 |
|  | SEP | 0.96 | 0.54 | 0.30 | 0.58 | 0.35 | 1.36 | 0.11 |
|  | OCT | 27.57 | 8.23 | 11.93 | 35.46 | 13.37 | 49.48 | 1.44 |
|  | NOV | 19.93 | 5.46 | 12.78 | 35.38 | 11.70 | 63.62 | 1.07 |
|  | DEC | 12.52 | 15.31 | 9.34 | 22.75 | 29.83 | 28.35 | 1.37 |
|  | JAN | 5.16 | 13.10 | 4.19 | 8.50 | 12.10 | 11.60 | 0.23 |
|  | FEB | 0.25 | 2.01 | 0.44 | 0.82 | 1.52 | 1.13 | 0.03 |
|  | MAR | 5.52 | 5.80 | 4.52 | 20.85 | 13.85 | 4.57 | 0.00 |
| 2010./2011 | APR | 6.27 | 6.88 | 9.22 | 44.12 | 23.22 | 11.69 | 0.00 |
|  | MAY | 2.69 | 1.54 | 4.97 | 22.64 | 12.94 | 4.15 | 0.00 |
|  | JUNE | 0.78 | 1.23 | 1.62 | 0.45 | 3.56 | 0.66 | 8.84 |
|  | JULY | 0.81 | 0.97 | 1.07 | 0.33 | 2.70 | 0.61 | 6.55 |
|  | AUG | 0.22 | 0.74 | 0.66 | 0.15 | 1.29 | 0.17 | 3.12 |
|  | SEP | 0.65 | 0.12 | 0.15 | 0.42 | 0.08 | 0.73 | 0.03 |
|  | OCT | 6.50 | 2.23 | 3.23 | 10.18 | 1.53 | 14.10 | 0.36 |
|  | NOV | 18.53 | 3.52 | 5.81 | 15.70 | 3.88 | 27.31 | 1.03 |
|  | DEC | 44.73 | 20.23 | 27.11 | 113.92 | 23.30 | 100.87 | 0.00 |
|  | JAN | 2.15 | 7.12 | 3.20 | 12.40 | 3.29 | 10.74 | 0.00 |
|  | FEB | 2.16 | 1.57 | 1.93 | 7.36 | 1.39 | 6.30 | 0.00 |

**Supplementary Data 1** Biogeochemical particle flux monthly average (Bsi, Biosilica; POC, Particle Organic Carbon; PIC, Particle Inorganic Carbon) and zooplankton calcifies flux (PT, Pteropods; FOR, Foraminifera; COCC, Coccolithophores; OST, Ostracodes) expressed as mg*m^-2^*d^-1^, at P2 and P3 during the austral summer 2009/2010 and 2010/2011.
