# Supplementary material for: Threatened species drive the strength of the carbonate pump in the northern Scotia Sea
Source: Nat Commun. 2018 Nov 2;9:4592. doi: 10.1038/s41467-018-07088-y (PMC6214935; doi:10.1038/s41467-018-07088-y)
Supplement: Supplementary file 4 — Supplementary Data 2 [file 41467_2018_7088_MOESM4_ESM.docx]

**Supplementary Data 2**

**Threatened species drive the strength of the carbonate pump in the Scotia Sea (Southern Ocean)**

Manno et al.

| P2 | mg/m2/d  PIC | Ψ | Fd  POC | depth. | WML | b | GROSS  FWLD (POC) | FWLD (PIC) | NET | gross-net | (gross-net)gross*100  %CCP |
| --- | --- | --- | --- | --- | --- | --- | --- | --- | --- | --- | --- |
| autmn | 1.16 | 0.75 | 1.35 | 1500 | 200 | 1.43 | 24.09 | 0.87 | 23.22 | 0.87 | 3.62 |
| winter | 0.26 | 0.75 | 0.81 | 1500 | 200 | 1.43 | 14.41 | 0.19 | 14.22 | 0.19 | 1.35 |
| spring | 0.68 | 0.75 | 1.22 | 1500 | 200 | 1.43 | 21.76 | 0.51 | 21.25 | 0.51 | 2.35 |
| summer | 3.25 | 0.75 | 3.28 | 1500 | 200 | 1.43 | 58.47 | 2.43 | 56.03 | 2.43 | 4.16 |
| autmn | 3.94 | 0.75 | 0.76 | 1500 | 200 | 1.43 | 13.56 | 2.95 | 10.60 | 2.95 | 21.80 |
| winter | 3.09 | 0.75 | 0.87 | 1500 | 200 | 1.43 | 15.52 | 2.31 | 13.20 | 2.31 | 14.91 |
| spring | 3.94 | 0.75 | 1.11 | 1500 | 200 | 1.43 | 19.77 | 2.95 | 16.82 | 2.95 | 14.94 |
| summer | 8.82 | 0.75 | 1.91 | 1500 | 200 | 1.43 | 34.11 | 6.62 | 27.50 | 6.62 | 19.40 |
| P3 |  |  |  |  |  |  |  |  |  |  |  |
| autmn | 0.57 | 0.75 | 1.02 | 2000 | 200 | 0.78 | 6.17 | 0.43 | 5.74 | 0.43 | 6.97 |
| winter | 0.71 | 0.75 | 0.80 | 2000 | 200 | 0.78 | 4.81 | 0.53 | 4.28 | 0.53 | 11.08 |
| spring | 9.76 | 0.75 | 7.39 | 2000 | 200 | 0.78 | 44.54 | 7.32 | 37.23 | 7.32 | 16.43 |
| summer | 3.65 | 0.75 | 10.14 | 2000 | 200 | 0.78 | 61.11 | 2.74 | 58.37 | 2.74 | 4.48 |
| autmn | 8.34 | 0.75 | 4.74 | 2000 | 200 | 0.78 | 28.56 | 6.26 | 22.30 | 6.26 | 21.91 |
| winter | 1.12 | 0.75 | 0.97 | 2000 | 200 | 0.78 | 5.83 | 0.84 | 4.99 | 0.84 | 14.42 |
| spring | 16.54 | 0.75 | 11.41 | 2000 | 200 | 0.78 | 68.75 | 12.41 | 56.34 | 12.41 | 18.05 |
| summer | 9.83 | 0.75 | 9.64 | 2000 | 200 | 0.78 | 58.08 | 7.37 | 50.71 | 7.37 | 12.70 |
|  |  |  |  |  |  |  |  |  |  |  |  |
| P2 | mg/m2/d  Pteropod | Ψ | Fd  POC | depth. | WML | b | GROSS  FWLD (POC) | FWLD (PIC) | NET | gross-net | (gross-net)gross*100  %CCP |
| autmn | 0.39 | 0.75 | 1.35 | 1500 | 200 | 1.43 | 24.09 | 0.29 | 23.80 | 0.29 | 1.20 |
| winter | 0.03 | 0.75 | 0.81 | 1500 | 200 | 1.43 | 14.41 | 0.02 | 14.39 | 0.02 | 0.16 |
| spring | 0.10 | 0.75 | 1.22 | 1500 | 200 | 1.43 | 21.76 | 0.08 | 21.69 | 0.08 | 0.36 |
| summer | 1.02 | 0.75 | 3.28 | 1500 | 200 | 1.43 | 58.47 | 0.77 | 57.70 | 0.77 | 1.31 |
| autmn | 3.39 | 0.75 | 0.97 | 1500 | 200 | 1.43 | 17.26 | 2.54 | 14.72 | 2.54 | 14.72 |
| winter | 1.19 | 0.75 | 1.30 | 1500 | 200 | 1.43 | 23.19 | 0.89 | 22.30 | 0.89 | 3.85 |
| spring | 1.91 | 0.75 | 1.71 | 1500 | 200 | 1.43 | 30.52 | 1.43 | 29.09 | 1.43 | 4.68 |
| summer | 5.01 | 0.75 | 1.81 | 1500 | 200 | 1.43 | 32.33 | 3.76 | 28.57 | 3.76 | 11.62 |
| P3 |  |  |  |  |  |  |  |  |  |  |  |
| autmn | 0.09 | 0.75 | 1.02 | 2000 | 200 | 0.78 | 6.17 | 0.07 | 6.11 | 0.07 | 1.07 |
| winter | 0.07 | 0.75 | 0.80 | 2000 | 200 | 0.78 | 4.81 | 0.06 | 4.76 | 0.06 | 1.15 |
| spring | 2.97 | 0.75 | 7.39 | 2000 | 200 | 0.78 | 44.54 | 2.23 | 42.32 | 2.23 | 5.00 |
| summer | 0.92 | 0.75 | 10.14 | 2000 | 200 | 0.78 | 61.11 | 0.69 | 60.41 | 0.69 | 1.14 |
| autmn | 4.62 | 0.75 | 4.74 | 2000 | 200 | 0.78 | 28.56 | 3.46 | 25.10 | 3.46 | 12.12 |
| winter | 0.04 | 0.75 | 0.97 | 2000 | 200 | 0.78 | 5.83 | 0.03 | 5.81 | 0.03 | 0.48 |
| spring | 5.68 | 0.75 | 11.41 | 2000 | 200 | 0.78 | 68.75 | 4.26 | 64.49 | 4.26 | 6.20 |
| summer | 4.75 | 0.75 | 9.64 | 2000 | 200 | 0.78 | 58.08 | 3.56 | 54.52 | 3.56 | 6.14 |
|  |  |  |  |  |  |  |  |  |  |  |  |
| P2 | mg/m2/d  Coccolith. | Ψ | Fd  POC | depth. | WML | b | GROSS  FWLD (POC) | FWLD (PIC) | NET | gross-net | (gross-net)gross*100  %CCP |
| autmn | 0.17 | 0.75 | 1.35 | 1500 | 200 | 1.43 | 24.09 | 0.13 | 23.97 | 0.13 | 0.52 |
| winter | 0.01 | 0.75 | 0.81 | 1500 | 200 | 1.43 | 14.41 | 0.01 | 14.40 | 0.01 | 0.06 |
| spring | 0.41 | 0.75 | 1.22 | 1500 | 200 | 1.43 | 21.76 | 0.30 | 21.46 | 0.30 | 1.40 |
| summer | 0.86 | 0.75 | 3.28 | 1500 | 200 | 1.43 | 58.47 | 0.64 | 57.82 | 0.64 | 1.10 |
| autmn | 0.89 | 0.75 | 1.03 | 1500 | 200 | 1.43 | 18.40 | 0.67 | 17.73 | 0.67 | 3.64 |
| winter | 1.09 | 0.75 | 1.40 | 1500 | 200 | 1.43 | 24.97 | 0.82 | 24.15 | 0.82 | 3.28 |
| spring | 2.57 | 0.75 | 1.80 | 1500 | 200 | 1.43 | 32.12 | 1.93 | 30.20 | 1.93 | 5.99 |
| summer | 1.91 | 0.75 | 1.48 | 1500 | 200 | 1.43 | 26.42 | 1.43 | 24.99 | 1.43 | 5.41 |
| P3 |  |  |  |  |  |  |  |  |  |  |  |
| autmn | 0.14 | 0.75 | 1.02 | 2000 | 200 | 0.78 | 6.17 | 0.10 | 6.07 | 0.10 | 1.67 |
| winter | 0.09 | 0.75 | 0.80 | 2000 | 200 | 0.78 | 4.81 | 0.07 | 4.75 | 0.07 | 1.36 |
| spring | 5.40 | 0.75 | 7.39 | 2000 | 200 | 0.78 | 44.54 | 4.05 | 40.49 | 4.05 | 9.09 |
| summer | 1.29 | 0.75 | 10.14 | 2000 | 200 | 0.78 | 61.11 | 0.97 | 60.14 | 0.97 | 1.58 |
| autmn | 1.04 | 0.75 | 4.74 | 2000 | 200 | 0.78 | 28.56 | 0.78 | 27.78 | 0.78 | 2.72 |
| winter | 0.06 | 0.75 | 0.97 | 2000 | 200 | 0.78 | 5.83 | 0.04 | 5.79 | 0.04 | 0.72 |
| spring | 9.41 | 0.75 | 11.41 | 2000 | 200 | 0.78 | 68.75 | 7.06 | 61.69 | 7.06 | 10.27 |
| summer | 4.06 | 0.75 | 9.64 | 2000 | 200 | 0.78 | 58.08 | 3.05 | 55.03 | 3.05 | 5.25 |
|  |  |  |  |  |  |  |  |  |  |  |  |
| P2 | mg/m2/d  Forams | Ψ | Fd  POC | depth | WML | b | GROSS  FWLD (POC) | FWLD (PIC) | NET | gross-net | (gross-net)gross*100  %CCP |
| autmn | 0.58 | 0.75 | 1.35 | 1500 | 200 | 1.43 | 24.09 | 0.44 | 23.65 | 0.44 | 1.82 |
| winter | 0.21 | 0.75 | 0.81 | 1500 | 200 | 1.43 | 14.41 | 0.16 | 14.25 | 0.16 | 1.11 |
| spring | 0.16 | 0.75 | 1.22 | 1500 | 200 | 1.43 | 21.76 | 0.12 | 21.65 | 0.12 | 0.55 |
| summer | 1.33 | 0.75 | 3.28 | 1500 | 200 | 1.43 | 58.47 | 1.00 | 57.47 | 1.00 | 1.71 |
| autmn | 0.30 | 0.75 | 0.38 | 1500 | 200 | 1.43 | 6.80 | 0.23 | 6.58 | 0.23 | 3.31 |
| winter | 0.76 | 0.75 | 0.97 | 1500 | 200 | 1.43 | 17.25 | 0.57 | 16.69 | 0.57 | 3.28 |
| spring | 0.86 | 0.75 | 0.91 | 1500 | 200 | 1.43 | 16.21 | 0.64 | 15.56 | 0.64 | 6.54 |
| summer | 0.59 | 0.75 | 0.71 | 1500 | 200 | 1.43 | 12.71 | 0.44 | 12.26 | 0.44 | 5.36 |
| P3 |  |  |  |  |  |  |  |  |  |  |  |
| autmn | 0.33 | 0.75 | 1.02 | 2000 | 200 | 0.78 | 6.17 | 0.25 | 5.92 | 0.25 | 4.02 |
| winter | 0.54 | 0.75 | 0.80 | 2000 | 200 | 0.78 | 4.81 | 0.40 | 4.41 | 0.40 | 8.35 |
| spring | 1.19 | 0.75 | 7.39 | 2000 | 200 | 0.78 | 44.54 | 0.89 | 43.65 | 0.89 | 2.01 |
| summer | 1.40 | 0.75 | 10.14 | 2000 | 200 | 0.78 | 61.11 | 1.05 | 60.06 | 1.05 | 1.72 |
| autmn | 2.69 | 0.75 | 4.74 | 2000 | 200 | 0.78 | 28.56 | 2.02 | 26.54 | 2.02 | 7.06 |
| winter | 0.30 | 0.75 | 0.97 | 2000 | 200 | 0.78 | 5.83 | 0.22 | 5.61 | 0.22 | 3.80 |
| spring | 1.12 | 0.75 | 11.41 | 2000 | 200 | 0.78 | 68.75 | 0.84 | 67.91 | 0.84 | 1.22 |
| summer | 1.01 | 0.75 | 9.64 | 2000 | 200 | 0.78 | 58.08 | 0.76 | 57.32 | 0.76 | 1.31 |
|  |  |  |  |  |  |  |  |  |  |  |  |
| P2 | mg/m2/d  ostracods | Ψ | Fd  POC | depth | WML | b | GROSS  FWLD (POC) | FWLD (PIC) | NET | gross-net | (gross-net)gross*100  %CCP |
| autmn | 0.02 | 0.75 | 1.35 | 1500 | 200 | 1.43 | 24.09 | 0.02 | 24.08 | 0.02 | 0.07 |
| winter | 0.03 | 0.75 | 0.81 | 1500 | 200 | 1.43 | 14.41 | 0.02 | 14.39 | 0.02 | 0.16 |
| spring | 0.01 | 0.75 | 1.22 | 1500 | 200 | 1.43 | 21.76 | 0.01 | 21.75 | 0.01 | 0.05 |
| summer | 0.03 | 0.75 | 3.28 | 1500 | 200 | 1.43 | 58.47 | 0.02 | 58.44 | 0.02 | 0.04 |
| autmn | 0.06 | 0.75 | 1.03 | 1500 | 200 | 1.43 | 18.40 | 0.04 | 18.35 | 0.04 | 0.24 |
| winter | 1.55 | 0.75 | 1.40 | 1500 | 200 | 1.43 | 24.97 | 1.16 | 23.81 | 1.16 | 4.65 |
| spring | 0.06 | 0.75 | 2.41 | 1500 | 200 | 1.43 | 42.96 | 0.04 | 42.92 | 0.04 | 0.10 |
| summer | 0.92 | 0.75 | 2.81 | 1500 | 200 | 1.43 | 50.17 | 0.69 | 49.48 | 0.69 | 1.37 |
| P3 |  |  |  |  |  |  |  |  |  |  |  |
| autmn | 0.02 | 0.75 | 1.02 | 2000 | 200 | 0.78 | 6.17 | 0.01 | 6.16 | 0.01 | 0.21 |
| winter | 0.01 | 0.75 | 0.80 | 2000 | 200 | 0.78 | 4.81 | 0.01 | 4.80 | 0.01 | 0.22 |
| spring | 0.20 | 0.75 | 7.39 | 2000 | 200 | 0.78 | 44.54 | 0.15 | 44.40 | 0.15 | 0.33 |
| summer | 0.04 | 0.75 | 10.14 | 2000 | 200 | 0.78 | 61.11 | 0.03 | 61.08 | 0.03 | 0.04 |
| autmn | 0.00 | 0.75 | 4.74 | 2000 | 200 | 0.78 | 28.56 | 0.00 | 28.56 | 0.00 | 0.00 |
| winter | 0.73 | 0.75 | 0.97 | 2000 | 200 | 0.78 | 5.83 | 0.55 | 5.29 | 0.55 | 9.42 |
| spring | 0.33 | 0.75 | 11.41 | 2000 | 200 | 0.78 | 68.75 | 0.25 | 68.50 | 0.25 | 0.36 |
| summer | 0.00 | 0.75 | 9.64 | 2000 | 200 | 0.78 | 58.08 | 0.00 | 58.08 | 0.00 | 0.00 |

**Supplementary Data 2** Calculation of reduction of the CO_2_ drawdown by the biological pump due to CO_2_ production during the calcification process expressed as (CCpump,%)=(PICflux**Ψ*)/POC_WLM_flux*100 , where : Ψ , mole of CO_2_ emitted by a mole of CO_3_^2+^ precipitated during the calcification process; PIC is the CaCO_3_ flux measured at the sediment trap (taken as a minimum estimate of PIC flux at the base of the winter mixed layer); POC_WLM_ flux is the POC flux measured at the sediment trap where the deployment depth were normalized to the base of the winter mixed layer (200 m) using the expression: F_WML_=F_d_ (WML/d)^b^; Fd is the flux at the sediment trap deployment depth, d is the sediment trap deployment depth, WML is 200m and the exponent b characterizes the attenuation of flux with depth in P2 and P3.
